# Fucosylated hemocyanin regulates ER stress to enhance shrimp antibacterial immunity

**DOI:** 10.1080/21505594.2026.2721775

**Published:** 2026-09-10

**Authors:** Mingming Jiang, Junyang Zhou, Xinyue Ren, Yiqi Liu, Jiawei Cheng, Yueling Zhang, Zhihong Zheng

**Affiliations:** Guangdong Provincial Key Laboratory of Marine Biotechnology, Institute of Marine Sciences, Shantou University, Shantou, China

**Keywords:** Fucosylation, Penaeus vannamei, ER stress, hemocyanin, antibacterial immunity

## Abstract

Invertebrates rely on post-translational modifications (PTMs) of immune effectors to regulate innate immunity, yet how hemocyanin fucosylation mediates intracellular antimicrobial signaling remains a critical knowledge gap in crustacean immunology. Although fucosylation enhances the in *vitro* antibacterial activity of *Penaeus vannamei* hemocyanin (*Pv*HMC), its function in regulating intracellular stress-immune crosstalk remains unclear. Here, we identify a novel axis linking *Pv*HMC fucosylation, endoplasmic reticulum stress (ERS), and antimicrobial peptide (AMP) production to bolster shrimp defense against *Vibrio parahaemolyticus*. Biotin pull-down coupled with LC-MS/MS revealed that highly fucosylated *Pv*HMC preferentially associates with the ER chaperone *Pv*Bip, and this fucosylation-associated interaction was further supported by GST pull-down assays. Functional assays showed that High-Fuc-*Pv*HMC enhanced a *Pv*Bip-*Pv*Xbp1s‑associated ER stress response and induced the transcription of key AMPs, including *Pv*PEN2, *Pv*LYZ2, *Pv*ALF2, and *Pv*ALF3. These effects were attenuated by the ER stress inhibitor 4-PBA. *In vivo*, High-Fuc-*Pv*HMC reduced hemolymph *Vibrio* loads and improved shrimp survival after *V. parahaemolyticus* challenge, whereas reduced *Pv*HMC fucosylation or ER stress inhibition weakened these protective effects. Collectively, our findings uncover an unrecognized intracellular role for hemocyanin whereby fucosylation enables *Pv*HMC to modulate ERS via *Pv*Bip-*Pv*Xbp1s signaling for AMP-driven antibacterial immunity. This study expands the functional repertoire of this canonical oxygen-transport and immune effector protein, and provides key mechanistic insights into PTM-mediated immune regulation in crustaceans.

## Introduction

Innate immunity serves as the first line of defense for invertebrates against invading pathogens, and its functional plasticity is critical for coping with the escalating environmental perturbations in aquatic ecosystems [1]. Climate change, ocean acidification, and anthropogenic pollution have degraded marine habitats globally, imposing severe physiological stress on crustaceans including *P. vannamei* (a species of immense aquacultural and ecological significance) and disrupting the homeostasis of their innate immune systems [2,3]. Unlike vertebrates, crustaceans lack vertebrate-like adaptive immune repertoires and therefore predominantly rely on conserved innate immune effectors and post-translational modification (PTM)-mediated functional diversification of these molecules to mount effective antimicrobial responses [4]. Uncovering the PTM-driven immunoregulatory mechanisms in crustaceans is therefore central to understanding crustacean immune regulation and developing strategies to mitigate disease outbreaks in aquaculture.

Post-translational modifications, including glycosylation, phosphorylation, and acetylation, are pivotal for expanding the functional repertoire of immune effectors by altering protein conformation, subcellular localization, and molecular interaction networks [4]. Among these modifications, fucosylation has emerged as a key modulator of immune cell signaling, pathogen recognition, and host–pathogen interactions across metazoans. It is catalyzed by fucosyltransferases (FUTs) and reversed by α-L-fucosidases (AFUs) [5–8]. For instance, pathogenic toxins (e.g. cholera toxin) exploit host cell surface fucosylated glycans to facilitate cellular entry and pathogenesis [9], while host-derived fucosylation of immune receptors enhances pathogen clearance by promoting ligand binding and downstream signal activation [7]. Despite these advances, the role of fucosylation in regulating intracellular immune signaling cascades in crustaceans remains largely unexplored.

Hemocyanin (HMC), a multifunctional glycoprotein abundant in arthropod and mollusk hemolymph, fulfills dual roles as an oxygen transporter and a core innate immune effector [10]. Beyond its respiratory function, HMC exhibits a broad spectrum of antimicrobial activities, including direct bacterial agglutination, viral neutralization, phenoloxidase-like activity, and proteolytic cleavage into antimicrobial peptides (AMPs) [11–18]. Moreover, HMC modulates immune signaling by interacting with components of the MAPK pathway to regulate AMP production [19], and links metabolic homeostasis to immune defense by controlling fatty acid metabolism via endoplasmic reticulum stress (ERS) [20]. The functional versatility of HMC is further shaped by PTMs, with ubiquitination, phosphorylation, and acetylation all shown to modulate its antibacterial activity in *P. vannamei* [21–23]. *Pv*FUT8/10-mediated fucosylation enhances *Pv*HMC binding to Gram-negative bacteria and increases its bactericidal capacity [24]. However, whether fucosylated *Pv*HMC regulates intracellular antimicrobial signaling beyond its direct antibacterial activity remains unknown.

The endoplasmic reticulum (ER) is a conserved organelle that maintains cellular proteostasis and integrates stress responses with immune signaling via the unfolded protein response (UPR), also referred to as endoplasmic reticulum stress (ERS) [25,26]. Because Bip-mediated protein-folding stress and Xbp1s activation are closely associated with UPR signaling in the rough ER, the ER stress discussed in this study primarily refers to UPR-related protein-folding stress rather than smooth ER-associated lipid metabolism or calcium storage. ERS is initiated by three transmembrane sensors (IRE1α, PERK, and ATF6) that are sequestered by the chaperone binding immunoglobulin protein (Bip) under basal conditions, whereas upon ER stress, Bip dissociates from these sensors to trigger downstream cascades regulating lipid metabolism, inflammatory responses, and AMP expression [27–30]. In vertebrates, ERS is a well-established regulator of innate immunity, but its interplay with PTM-modified immune effectors in invertebrates is poorly characterized. Notably, HMC has been linked to ERS-mediated metabolic regulation in shrimp [20], yet whether and how fucosylation of HMC modulates ERS to shape antimicrobial immunity has never been investigated.

Building on our previous finding that *Pv*HMC fucosylation enhances direct antibacterial activity, the present study investigates whether fucosylated *Pv*HMC also regulates intracellular immune signaling. We focus on the potential involvement of *Pv*Bip-*Pv*Xbp1s-associated ER stress signaling and AMP expression during *V. parahaemolyticus* challenge. By integrating proteomic screening, protein–protein interaction assays, pharmacological modulation of ER stress, and *in vivo* infection assays, this study aims to clarify how *Pv*HMC fucosylation contributes to shrimp antibacterial immunity beyond direct pathogen binding.

## Materials and methods

### Ethics statement

The present study was conducted in accordance with the Administration of Science and Technology Ethics Review (Trial, 2023, https://www.most.gov.cn/xxgk/xinxifenlei/fdzdgknr/fgzc/gfxwj/gfxwj2023/202310/t20231008_188309.html) and the Requirements of Laboratory Animal Welfare (NY/T 4834–2025, http://www.aqsc.agri.cn/zlbz/bzgg/pgazzp/202604/P020260401619818379005.pdf). Formal ethical approval is not required for experiments involving decapod crustaceans under the current local institutional regulations. Nevertheless, all procedures were designed to minimize animal stress and suffering. Shrimp were cold-anesthetized on ice before hemolymph collection. After hemolymph withdrawal, sampled animals were euthanized immediately by prolonged ice exposure to ensure death. During infection experiments, shrimp were monitored regularly, and moribund individuals showing loss of equilibrium, severe lethargy, or lack of response to mechanical stimulation were removed and humanely euthanized. All efforts were made to reduce the number of animals used while ensuring adequate biological replication.

### Experimental animals, tissue collection, and immune challenge

Pacific white shrimp (*P. vannamei*, average body weight: 10 ± 2 g, mixed-sex) were obtained from Shantou Huaxun Aquatic Product Co., Ltd. (Guangdong, China), a commercial aquaculture facility with standardized pathogen-free breeding protocols. Upon arrival, shrimp were acclimated in recirculating aerated artificial seawater systems (salinity: 30 ‰, temperature: 25 ± 0.5°C, pH: 7.8–8.0) for 2–3 d before experimentation. During acclimation, shrimp were fed a commercial pelleted diet (crude protein ≥40%, crude lipid ≥8%) once daily at 5% of body weight, with 50% water exchange performed daily to maintain water quality. Approximately 2,000 *Penaeus vannamei* were used across all experiments in this study. For the experimental group with an average body weight of 10 ± 2 g, the *n*-value was 40.

For tissue and hemolymph collection, three randomly selected shrimp were anesthetized on ice for 5 min to minimize stress. The three shrimp in each biological replicate were randomly selected from all surviving individuals in the corresponding subgroup at the experimental endpoint, with consistent body weight and health status. Hemolymph was aspirated from the ventral sinus of the cephalothorax using a sterile 1 mL syringe preloaded with ice-cold anticoagulant buffer (7.576 g/L sodium citrate, 6.304 g/L citric acid, 19.818 g/L D-glucose, 8.182 g/L NaCl, pH 6.0) at a 1:1 (v/v) ratio of hemolymph to buffer. A 0.7 mm (22 G) disposable sterile needle (0.7 × 30TW.LB) was used for the extraction, which is well suited for routine shrimp hemolymph collection. All hemolymph samples were collected from randomly selected shrimp at the end of the *in vivo* experiment. Each shrimp was euthanized immediately after sampling to ensure that no individual was bled repeatedly. Hemocytes were isolated by centrifugation at 800 × g for 10 min at 4°C, and the resulting cell pellet was immediately processed or snap-frozen in liquid nitrogen and stored at −80°C for subsequent RNA/protein extraction.

For the *V. parahaemolyticus* infection challenge, shrimp were randomly allocated into experimental groups to avoid selection bias, and all infection assays were performed in three independent biological replicates to ensure reproducibility. Briefly, shrimp in the pathogen-challenged group were continuously maintained in aerated seawater containing *V. parahaemolyticus* (1 × 10^6^ CFU/mL, a virulent isolate from diseased shrimp) throughout the entire experimental period, while control shrimp were kept in sterile normal seawater without bacterial exposure. All other environmental conditions (temperature, salinity, feeding, water exchange) remained identical between the challenged and control groups.

### Antibodies and lectins

The following antibodies and working concentrations were used: Aleuria Aurantia Lectin (AAL) (Cat#1395–1, Vectorlabs, America, 1:3000 for Lectin blot), Mouse anti-GST antibody (Cat#HT601-01, 1:1000, TransGen Biotech, Beijing, China, 1:1000 for Western blot), Rabbit anti-Bip antibody (Cat#AB310, Beyotime Biotechnology, Shanghai, China, 1:1000 for Western blot), Rabbit anti-Xbp1s antibody (Cat# AF8366, Beyotime Biotechnology, Shanghai, China, 1:1000 for Western blot), Horseradish peroxidase (HRP)-linked goat anti-rabbit (Cat#31460, Thermo Fisher Scientific, Waltham, MA, USA, 1:3000, for Western blot), and HRP-linked goat anti-mouse (Cat#A32723, Thermo Fisher Scientific, Waltham, MA, USA, 1:3000 for Western blot).

### RNA extraction, cDNA synthesis, and quantitative reverse-transcription PCR (RT-qPCR)

Total RNA was extracted from shrimp hemocytes and tissues using the RNA Fast 200 Total RNA Extraction Kit (Feijie Biotech, Shanghai, China), with on-column DNase I digestion (included in the kit) to eliminate genomic DNA contamination. RNA purity and concentration were assessed using a NanoDrop 2000 spectrophotometer (Thermo Fisher Scientific) (A_260_/A_280_ ratio: 1.8–2.1) and 1% agarose gel electrophoresis (integrity verified by intact 18S and 28S rRNA bands).

First-strand cDNA was synthesized from 1 μg of high-quality total RNA using the EasyScript® One-Step gDNA Removal and cDNA Synthesis SuperMix (TransGen Biotech, Beijing, China), following the manufacturer’s protocol. The reaction was performed at 42°C for 15 min, with a final inactivation step at 85°C for 5 s; cDNA products were diluted 1:5 in nuclease-free water and stored at −20°C for RT-qPCR analysis.

Gene-specific primers (Table 1) were designed using Primer Premier 5.0 software (Premier Biosoft, Palo Alto, CA, USA) based on *P. vannamei* sequences from the NCBI GenBank database. Primer specificity was validated via melting‑curve analysis, with a single specific peak observed for each primer pair. RT-qPCR was performed in 20 μL reaction volumes on a LightCycler 480 II system (Roche Diagnostics, Basel, Switzerland), with the following reaction mix: 10 μL 2 × RealStar Green Power Mixture (GenStar Biotech, Beijing, China), 1 μL cDNA template (20 ng/μL), 1 μL each of forward/reverse primer (10 μM), and 7 μL nuclease-free water. Thermal cycling conditions were: initial denaturation at 95°C for 10 min; 45 cycles of 95°C for 15 s, 60°C for 30 s (annealing/extension); and a final melting‑curve analysis (60–95°C, 0.1°C/s increment) to confirm amplicon specificity. Relative gene expression was calculated using the 2^−ΔΔCt^ method [31], with elongation factor 1α (EF1α) as the internal reference gene (stable expression verified across all experimental groups via geNorm analysis, *M* value < 0.5). All RT-qPCR reactions were performed in technical triplicate for each of three independent biological replicates.

**Table 1.** Primer sequences used in this study.

Primer name	Sequence(5”−3”)
**Gene cloning**	
pGEX-6P-1-*Pv*Bip-F	TCCAGGGGCCCCTGGGATCCATGAGGTGTTGGACTGCATTAG
pGEX-6P-1-*Pv*Bip-R	CCCGGGAATTCCGGGGATCCCTACAATTCGTCCTTTTCATAA
**Real-time qPCR**	
*Pv*AFU-qF	CGTCAAGGATACGGTGGTC
*Pv*AFU-qR	CGAGGGTGGTGATGAGGTC
*Pv*FUT8-qF	CGGCTGGCGGTACAACAA
*Pv*FUT8-qR	ACGGTCCGAGATGTCCCTG
*Pv*FUT10-qF	ACAAGTATGTCAGTGAATTAGCCA
*Pv*FUT10-qR	CTCGGTGATGTAGTCGTCGC
*Pv*EF-1α-qF	TATGCTCCTTTTGGACGTTTTGC
*Pv*EF-1α-qR	CCTTTTCTGCGGCCTTGGTAG
*Pv*Bip-qF	GAGCGTCTGATTGGTGATT
*Pv*Bip-qR	GTGGCTTGTCGTTCTTGTT
*Pv*Xbp1s-qF	AACTACGGGACCTGACATCTGC
*Pv*Xbp1s-qR	ACTGCCTTCTGCTGATCCACC
*Pv*ALF1-qF	CAGCGGCCCTCCTTCTATAG
*Pv*ALF1-qR	TCGTCCTCCGTGATGAGATTACT
*Pv*ALF2-qF	GCCATTGCGAACAAACTCACT
*Pv*ALF2-qR	CACATGCGACCCCTGAAATAC
*Pv*ALF3-qF	GCTGTGGAGGAACGAGGAGA
*Pv*ALF3-qR	TGATGTAAGGTTTGACGGTGAAG
*Pv*ALF4-qF	ACTGGCGAGAGGGAGATGTTT
*Pv*ALF4-qR	CACGTTGCTCAGGGTTGGA
*Pv*PEN2-qF	ACCACCGTTCAGACCTGTTTG
*Pv*PEN2-qR	TTCTCCGTCAATTTCTTTATCCTTT
*Pv*PEN3-qF	GCCTATTGGTCCATACAACGG
*Pv*PEN3-qR	GTTTTCATCGTGTTCTCCGTCA
*Pv*PEN4-qF	GCCCGTTACCCAAACCATC
*Pv*PEN4-qR	TCTTCTCCATCAACCAGACTATCC
*Pv*LYZ1-qF	GGTGACTGGGACTGCAACG
*Pv*LYZ1-qR	TGGAGGTGGTGTGAAAAGCC
*Pv*LYZ2-qF	AGTACTGGTGCGGAAGCGAC
*Pv*LYZ2-qR	AGAACGGGAAGACAGAGTTGGA
*Pv*LYZ3-qF	CTCAAGCAACTGCTCCATGC
*Pv*LYZ3-qR	TCTTCTACGCATTTGTAAAAGTCG
*Pv*LYZ4-qF	GAGGAGGTAGTCGGCGTCAG
*Pv*LYZ4-qR	ACGTGTGGAACAGGCTGAAAA
*Pv*CRU1-qF	GCCCACGAACCAGAGACAC
*Pv*CRU1-qR	CCTGCGATCCGAAGAATGA
*Pv*CRU2-qF	TCGCTTAGGAGGAGGATTCG
*Pv*CRU2-qR	TAATTGCAGTTGAATCCGCCT
*Pv*CRU3-qF	TCTGGTCGTGTTGGTCTTGGT
*Pv*CRU3-qR	GACGTCGCTCGTATCTGGG
*Vibrio*-qF	GGCGTAAAGCGCATGCAGGT
*Vibrio*-qR	GAAATTCTACCCCCCTCTACAG
16S-qF	TGGAGCATGTGGTTTAATTCGA
16S-qR	TGCGGGACTTAACCCAACA
***In vitro*** **dsRNA synthesis**	
dsEGFP-T7F	GGATCCTAATACGACTCACTATAGGCGTAAACGGCCACAAGTT
dsEGFP-R	TTCACCTTGATGCCGTTC
dsEGFP-T7R	GGATCCTAATACGACTCACTATAGGTTCACCTTGATGCCGTTC
dsEGFP-F	CGTAAACGGCCACAAGTT
ds*Pv*AFU-T7F	GGATCCTAATACGACTCACTATAGG GCTCACCCCTGGAGTTTG
ds*Pv*AFU-R	CTGGATTCCTGCCCTTACA
ds*Pv*AFU-T7R	GGATCCTAATACGACTCACTATAGG CTGGATTCCTGCCCTTACA
ds*Pv*AFU-F	GCTCACCCCTGGAGTTTG
ds*Pv*FUT8-T7F	GGATCCTAATACGACTCACTATAGGCAAACCGCAGCCCAAGACA
ds*Pv*FUT8-R	GGACGAGAACGTGCAAACCA
ds*Pv*FUT8-T7R	GGATCCTAATACGACTCACTATAGGGGACGAGAACGTGCAAACCA
ds*Pv*FUT8-F	CAAACCGCAGCCCAAGACA
ds*Pv*FUT10-T7F	GGATCCTAATACGACTCACTATAGGGGGAGGCGGTCGTCTTGT
ds*Pv*FUT10-R	AGTGGTTTTTTTCCTTTGTGGG
ds*Pv*FUT10-T7R	GGATCCTAATACGACTCACTATAGGAGTGGTTTTTTTCCTTTGTGGG
ds*Pv*FUT10-F	GGGAGGCGGTCGTCTTGT
ds*Pv*Xbp1s-T7F	TAATACGACTCACTATAGGAGGAAGAGGCAGAGACT
dsPvXbp1s-R	GAGAGCAGAGTGTTGAGAG
ds*Pv*Xbp1s-T7R	TAATACGACTCACTATAGGAGAGCAGAGTGTTGAGAG
dsPvXbp1s-F	GAGGAAGAGGCAGAGACT

### Western blot and lectin blot

Total protein was extracted from hemocytes using RIPA lysis buffer (Beyotime Biotechnology) supplemented with 1 mM phenylmethylsulfonyl fluoride (PMSF) and 1× protease inhibitor cocktail (Roche Diagnostics) to prevent protein degradation. Protein concentration was quantified via BCA assay (Thermo Fisher Scientific), with 30 μg of total protein per lane mixed with 5× SDS loading buffer (42 mM Tris-HCl, 10% glycerol, 2.3% SDS, 5% 2-mercaptoethanol, 0.02% bromophenol blue, pH 6.8) and denatured at 100°C for 10 min.

Denatured proteins were separated by 10% SDS-PAGE (5% stacking gel, 10% separating gel) at 80 V for 30 min (stacking gel) and 120 V for 90 min (separating gel), then transferred to PVDF membranes (MilliporeSigma, Burlington, MA, USA, Cat# R0NB30936) using a Mini Trans-Blot Electrophoretic Transfer Cell (Bio-Rad Laboratories, Hercules, CA, USA, Cat# 1,658,030) at 100 V for 90 min at 4°C. Membranes were blocked with 5% nonfat dry milk in TBST (20 mM Tris-HCl, 150 mM NaCl, 0.1% Tween-20, pH 7.6) for 1 h at room temperature (RT), then incubated with primary antibodies or AAL lectin at 4°C overnight with gentle shaking. After three 10-min washes with TBST, membranes were incubated with HRP-conjugated secondary antibodies for 1 h at RT, followed by three additional TBST washes. Protein bands were visualized using an ECL Plus Chemiluminescent Substrate Kit (Thermo Fisher Scientific) and imaged on a ChemiDoc XRS+ Imaging System (Bio-Rad Laboratories). Band intensity was quantified using ImageJ software (v1.53t, NIH, USA), with β-actin as the loading control for normalization. For lectin blots, streptavidin-HRP (1:3000, Thermo Fisher Scientific) was used for signal detection instead of secondary antibodies.

### Biotin conjugation of hemocyanin

*Pv*HMC samples with varying fucosylation levels (High-Fuc-HMC, Mid-Fuc-HMC, Low-Fuc-HMC; 2 μg/mL, 500 μL per sample) were transferred to 1.5 mL centrifuge tubes. Biotin conjugation was performed using a EZ-Link™ Sulfo-NHS-LC-Biotin Kit (Thermo Fisher Scientific) per the manufacturer’s protocol: 250 μL biotin conjugation buffer and 250 μL freshly prepared activated biotin solution (reconstituted in dedicated dilution buffer immediately before use) were added to each sample, with incubation at RT for 2 h under end-over-end rotation (30 rpm).

Unconjugated biotin was removed using Zeba™ Spin Desalting Columns (7K MWCO, Thermo Fisher Scientific). Columns were pre-conditioned by centrifugation at 3000 rpm for 1 min at 4°C, followed by two additional centrifugations (3000 rpm, 2 min, 4°C) after removing column caps. Columns were equilibrated with 25 mL bioconjugation buffer (added in 5 mL aliquots, with centrifugation at 3000 rpm for 2 min after each aliquot). Conjugated protein samples were loaded onto the column resin bed, and purified biotinylated *Pv*HMC was collected via centrifugation (3000 rpm, 8 min, 4°C). Protein concentration was quantified via BCA assay, and conjugation efficiency was verified by dot blot using streptavidin-HRP (1:3000).

### Biotin pull-down

Streptavidin-coated magnetic beads (50 μL, Thermo Fisher Scientific) were transferred to a 1.5 mL low-protein-binding centrifuge tube (Eppendorf, Hamburg, Germany) and washed twice with 1 mL 1× wash buffer (0.01 M PBS, 0.1% Triton X-100, pH 7.4) on a magnetic rack to remove the storage buffer. Biotin-labeled fucosylated hemocyanin (*Pv*HMC) samples (High-Fuc-HMC, Mid-Fuc-HMC, Low-Fuc-HMC; 50 μg each, prepared as described in Biotin conjugation of Hemocyanin) were added to the beads, with the final volume adjusted to 1 mL using PBS, and incubated at RT for 30 min with end-over-end rotation (30 rpm). Unbound protein was retained for subsequent analysis to confirm binding efficiency. The bead-protein complexes were washed five times with 1 mL 1× wash buffer, then incubated with 1.5 mg of shrimp hemocyte lysate (prepared in RIPA buffer) at 4°C for 4 h with rotation. After five additional washes with wash buffer, bound protein complexes were eluted with 25 μL elution buffer (10 mM EDTA, 95% formamide, pH 8.2) at 37°C for 10 min with shaking (600 rpm). The eluate was collected via magnetic separation, mixed with 5× SDS loading buffer, denatured at 100°C for 10 min, and stored at −20°C for SDS-PAGE and silver staining analysis.

### Silver staining

Eluted protein samples from the biotin pull-down assay were separated by 5% stacking/10% separating SDS-PAGE as described in Western blot and lectin blot. After electrophoresis, gels were fixed overnight in Fixation Solution 1 (50% methanol, 10% acetic acid, 40% water) at RT with gentle shaking, followed by a 30-min incubation in Fixation Solution 2 (30% methanol, 10% acetic acid, 60% water). Gels were rinsed three times with nuclease-free water (10 min per rinse), then sensitized in 0.02% sodium thiosulfate for 10 min at RT with shaking. After three 10-min water rinses, gels were stained in the dark with 0.1% silver nitrate solution for 10 min, then briefly rinsed three times with water (≤1 min total rinse time to avoid over-destaining). Gels were developed in fresh developer solution (0.2% sodium carbonate, 0.04% formaldehyde) for ~2 min until protein bands were clearly visible. The reaction was terminated with stop solution (10% acetic acid) for 30 min. Stained gels were rinsed with water and imaged using an EPSON Expression 11,000 XL Scanner (Epson, Suwa, Japan) at 600 dpi resolution.

### RNA interference (RNAi)

Double-stranded RNAs (dsRNAs) targeting *Pv*FUT8, *Pv*AFU, *Pv*Xbp1s, and enhanced green fluorescent protein (EGFP, control) were synthesized using the T7 RiboMAX™ Express RNAi System (Promega, Madison, WI, USA), with gene-specific T7 promoter-tagged primers (Table 1). dsRNA integrity was verified by 1% agarose gel electrophoresis (single band of expected size), and concentration was quantified via NanoDrop 2000 before storage at −80°C.

For *in vivo* knockdown of *Pv*FUT8 and *Pv*AFU, acclimated shrimp were randomly assigned to three groups: dsEGFP, ds*Pv*FUT8, and ds*Pv*AFU. Shrimp in each group were intramuscularly injected with 10 μg of the corresponding dsRNA into the third abdominal segment. At 48 h post-injection, knockdown efficiency was evaluated by RT-qPCR, and the altered fucosylation status of purified *Pv*HMC was verified by AAL lectin blot. Subsequent experiments were performed only when the group-level knockdown efficiency reached ≥70%.

For *in vivo* knockdown of *Pv*Xbp1s, acclimated shrimp were randomly assigned to two groups: dsEGFP and ds*Pv*Xbp1s. Shrimp in each group were intramuscularly injected with 10 μg of the corresponding dsRNA into the third abdominal segment. At 72 h post-injection, knockdown efficiency and the expression of *Pv*PEN2 were verified by RT-qPCR. Subsequent experiments were performed only when the group-level knockdown efficiency reached ≥70%.

### GST pull-down assay

Recombinant GST-tagged *Pv*Bip (GST-*Pv*Bip) and empty GST protein (control) were expressed in *Escherichia coli* BL21 (DE3) cells (TransGen Biotech) and purified using Glutathione Sepharose 4B resin (Cytiva, Marlborough, MA, USA) per standard protocols. Purified *Pv*HMC samples (High-Fuc, Mid-Fuc, Low-Fuc; 20 μg each) were incubated with GST-*Pv*Bip-bound or GST-bound resin (50 μL slurry) in 1 mL binding buffer (0.01 M PBS, 1% Triton X-100, pH 7.4) at 4°C for 2 h with end-over-end rotation. The resin was pelleted by centrifugation at 560 rpm for 1 min at RT, and the supernatant was discarded to remove unbound protein. The resin was washed 10 times with 500 μL binding buffer (2 min rotation per wash, followed by centrifugation at 500 × g for 1 min), then resuspended in 30 μL 1× SDS loading buffer and denatured at 100°C for 10 min. Eluted proteins were analyzed by SDS-PAGE and Western blot (Western blot and lectin blot), with GST antibody used to confirm bait protein immobilization and *Pv*HMC-specific antibody used to detect interacting target protein.

### Liquid chromatography-tandem mass spectrometry (LC-MS/MS) analysis

Differential protein bands (≈72 kDa, specific to the High-Fuc-HMC group) were excised from silver-stained SDS-PAGE gels under a laminar flow hood using sterile disposable scalpels, then transferred to enzyme-free 1.5 mL centrifuge tubes containing DEPC-treated water and shipped on ice to Shenzhen MicroNano Biotechnology Co., Ltd. (Shenzhen, China) for LC-MS/MS analysis.

Gel pieces were processed for mass spectrometry as follows: (1) rinsed three times with nuclease-free water and destained with 150 μL destaining solution (50% acetonitrile, 25 mM ammonium bicarbonate) until colorless; (2) dehydrated via sequential washes with 25 mM ammonium bicarbonate, 50% acetonitrile, and 100% acetonitrile (two washes each, 10 min per wash); (3) reduced with 50 μL 10 mM DTT at 56°C for 30 min, followed by alkylation with 50 μL 50 mM iodoacetamide (IAA) in the dark at RT for 15 min; (4) rehydrated with 15–20 μL trypsin solution (0.01 μg/μL, proteomics grade, Promega) on ice for 1 h, then overlaid with 30–40 μL 50 mM ammonium bicarbonate (10% acetonitrile) and digested at 37°C overnight; (5) peptide extraction with 100 μL extraction buffer (67% acetonitrile, 2% formic acid) at 37°C for 30 min, followed by 15 min of sonication; (6) pooled supernatants were concentrated to dryness via vacuum centrifugation and redissolved in 0.1% formic acid (mobile phase A).

Peptide separation was performed on an Easy nLC 1200 system (Thermo Fisher Scientific) using an Acclaim PepMap RSLC C18 column (75 μm × 25 cm, 2 μm, 100 Å) with a linear gradient of 5–38% mobile phase B (80% acetonitrile, 0.1% formic acid) over 30 min at a flow rate of 300 nL/min. Mass spectrometry was conducted on a Q Exactive Plus Orbitrap system (Thermo Fisher Scientific) with a Nano Flex ion source (spray voltage: 1.9 kV, capillary temperature: 275°C). Data-dependent acquisition (DDA) mode was used with the following parameters: full MS scans (m/z 350–2000) at 70,000 resolution, maximum injection time (MIT) = 100 ms; top 20 precursor ions (charge states 2+ to 5+) were selected for MS/MS scans at 17,500 resolution, MIT = 50 ms, higher-energy collisional dissociation (HCD) collision energy = 28 eV, dynamic exclusion = 25 s.

Raw MS data were processed using PEAKS Studio 8.5 (Bioinformatics Solutions Inc., Canada) and searched against the *P. vannamei* protein database (NCBI, accessed March 2024, 45,216 entries) with the following parameters: tryptic digestion (max 2 missed cleavages), precursor mass tolerance = 10 ppm, fragment mass tolerance = 0.05 Da, fixed modification (carbamidomethylation of Cys), variable modifications (oxidation of Met, deamidation of Asn/Gln, fucosylation of Asn). Protein identifications were accepted if they matched ≥2 unique peptides with a false discovery rate (FDR) <1%.

### In vivo *injection of PvHMC with distinct fucosylation levels*

To investigate the *in vivo* function of fucosylated *Pv*HMC, variants with distinct fucosylation levels were generated, purified, and validated prior to injection. Briefly, *Pv*HMC variants with different fucosylation levels were prepared according to the methods described in RNA interference (RNAi), and further purified by gel filtration chromatography using Sephadex G-200 medium (Solarbio LIFE SCIENCES, Beijing, China, Cat# S9160). Fractions corresponding to the major *Pv*HMC peak were collected, concentrated, and verified by SDS-PAGE/Coomassie staining and Western blot using anti-*Pv*HMC antibody. Equal amounts of purified *Pv*HMC were subjected to AAL lectin blot, and relative fucosylation levels were quantified by normalizing AAL signal intensity to total *Pv*HMC protein signal. Results were expressed as relative fold changes rather than percentage fucose coverage.

After verification, the purified *Pv*HMC preparations were used for *in vivo* injection. Shrimp were randomly allocated into four groups (*n* = 80 per group) and intramuscularly injected with 20 μg of recombinant EGFP (control), High-Fuc-HMC, Mid-Fuc-HMC, or Low-Fuc-HMC, respectively. At 12 h post-injection, hemolymph was collected from each group for subsequent functional analyses.

### Endoplasmic reticulum stress activation/inhibition assay

To induce ERS, tunicamycin (TM, Sangon Biotech, Shanghai, China) was dissolved in DMSO to prepare a 10 μg/μL stock solution (stored at 4°C for ≤1 week). Before injection, the stock was diluted to a working concentration of 6 ng/μL using sterile 0.01 M PBS (DMSO final concentration ≤0.1% to avoid solvent toxicity). Twenty healthy shrimp were randomly divided into two groups (*n* = 10 per group): the TM group received an intramuscular injection of 100 μL diluted TM solution, while the control group received 100 μL DMSO-PBS (0.1% DMSO). At 6 h post-injection, hemolymph was collected for RNA and protein extraction, with ERS induction verified via qPCR (*Pv*Bip, *Pv*Xbp1s expression) and Western blot (’Western blot and lectin blot’). TM was used only as a pharmacological positive control for ER stress activation and was not used to manipulate *Pv*HMC fucosylation.

For ERS inhibition, 4-phenylbutyric acid (4-PBA, MCE, Monmouth Junction, NJ, USA) was dissolved in DMSO to a 20 μg/μL stock solution (stored at −20°C). The stock was diluted to 0.1 μg/μL using sterile 0.01 M PBS containing 20% sulfobutylether-β-cyclodextrin (SRE-β-CD, to enhance solubility), with a control group receiving PBS-SRE-β-CD containing 0.1% DMSO. Shrimp injection and sample collection were performed as described for the TM assay, with ERS inhibition confirmed via reduced *Pv*Bip and *Pv*Xbp1s expression.

### Genomic DNA extraction and bacterial load analysis

To assess the impact of fucosylated *Pv*HMC and ERS on bacterial clearance, 1280 shrimp of uniform size were randomly allocated to two primary groups (*n* = 640 per group): Group A (pathogen challenge) was subjected to immersion infection with *V. parahaemolyticus* (1 × 10^6^ CFU/mL, a virulent isolate from diseased shrimp), while Group B (control) was cultured in normal seawater without bacterial immersion. At 48 h post-challenge, each primary group was subdivided into four subgroups (*n* = 160 per subgroup) and injected with 20 μg of EGFP (control), High-Fuc-HMC, Mid-Fuc-HMC, or Low-Fuc-HMC, respectively. Six hours later, each subgroup was further split into two sets (*n* = 80 per set): one set received ERS inhibitor 4-PBA (0.1 μg/μL, 100 μL/shrimp), and the other received an equal volume of sterile PBS, resulting in 16 total experimental groups (*n* = 80 per group).

For each subgroup, hemolymph was collected at 6 h post-4-PBA/PBS injection from five biological replicates, with three shrimp pooled per replicate, and bacterial pellets were obtained via sequential centrifugation (500 × g for 10 min at 4°C to pellet hemocytes, followed by 16,000 × g for 10 min at 4°C to pellet bacteria from the supernatant). Genomic DNA (gDNA) was extracted from bacterial pellets using the TIANamp Marine Animals DNA Kit (TIANGEN, Beijing, China) per the manufacturer’s protocol, with DNA purity verified by A260/A280 ratio (1.7–1.9). *V. parahaemolyticus* and total bacterial abundance were quantified via qPCR using species-specific (*Vibrio*-qF/qR) and universal 16S rRNA primers (Table 1), respectively. Standard curves for quantification were generated using serial dilutions of *V. parahaemolyticus* gDNA (10^2^–10^8^ copies/μL), with amplification efficiency confirmed to be 92–98%.

### Shrimp survival rate

To determine the cumulative mortality rate of shrimp under different treatments, the survival assay was performed in three independent biological replicates using the same experimental design described above. The number of dead shrimps in each group was recorded at 12-hour intervals up to 72 hours. The cumulative mortality rate was calculated using the Kaplan–Meier method [32] and statistical significance was assessed with the log-rank test [33] in GraphPad Prism (version 8.0.1).

### Prediction of putative Xbp1-binding sites

Putative Xbp1-binding motifs in the promoter regions of *P. vannamei* antimicrobial peptide (AMP) genes were predicted using the JASPAR database (https://jaspar.elixir.no/), a curated repository of transcription factor binding profiles. The 2000 bp upstream sequence of the *Pv*PEN2 transcription start site (GenBank Accession No.: AY351655.1) was retrieved from NCBI and analyzed using the JASPAR CORE vertebrate database (invertebrate profiles were used where available). Binding sites with a relative score > 0.8 (indicating high confidence) were selected for validation, with motif sequences and positions summarized in Table 2.

**Table 2.** Prediction of Xbp1s transcription factor-binding sites in AMP gene in *Penaeus vannamei.*

Sequence lD	Matrix ID	Score	Relative score	Predicted sequence	Motif graph
*Pv*PEN2	MA0414.1	9.75689	1.0000001	CTCGAGG	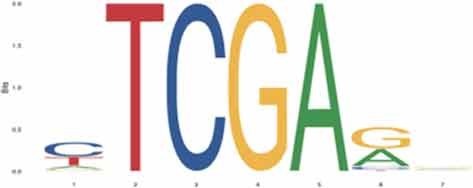
*Pv*PEN2	MA0414.2	9.45852	1	CTCGAG	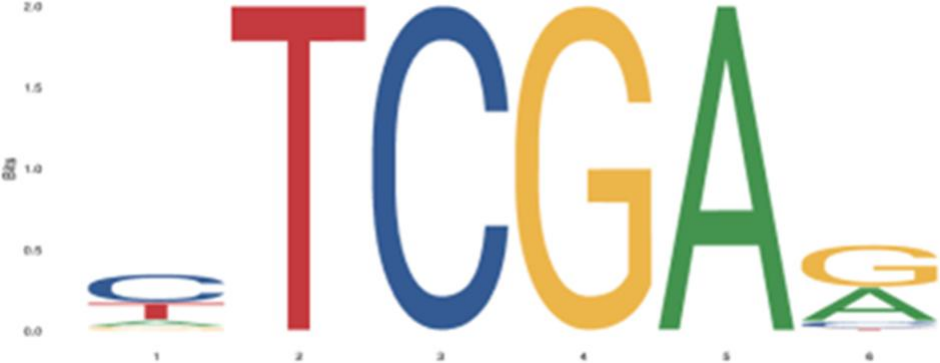
*Pv*PEN2	MA2293.1	6.22673	0.8285697	GGATGTGG	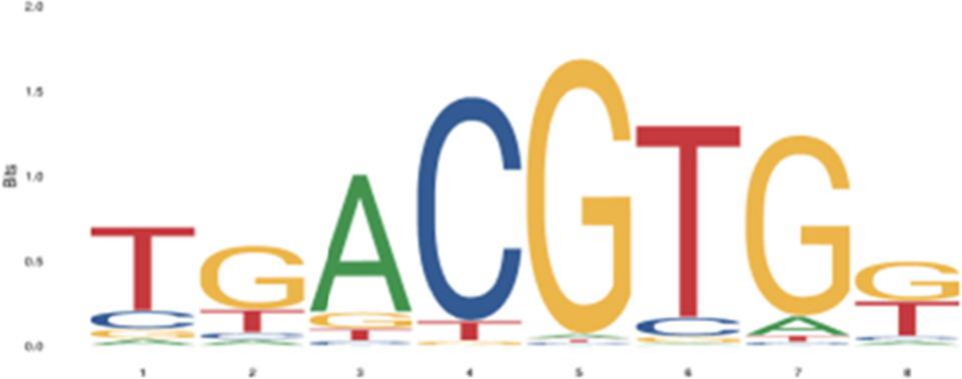
*Pv*PEN2	MA0414.1	4.61914	0.81723106	ATCGATA	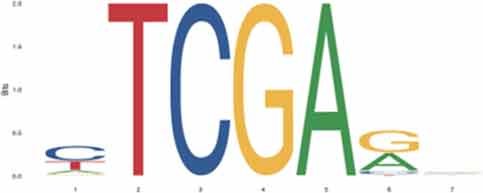
*Pv*PEN2	MA0414.1	4.40034	0.8094477	CACGAGG	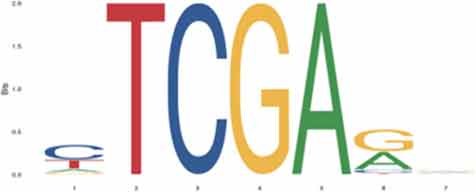

### Statistical analysis

Data are presented as mean ± SEM. Normality was assessed using the Shapiro–Wilk test, and homogeneity of variance was evaluated using Brown-Forsythe or Levene’s test. For comparisons between two groups, a two-tailed unpaired Student’s *t*-test was used when the data met parametric assumptions; otherwise, the Mann-Whitney U test was applied. For comparisons among three or more groups, one-way or two-way ANOVA followed by Tukey’s or Sidak’s multiple-comparison test was used as appropriate. For data with unequal variances, Welch’s ANOVA followed by Dunnett’s T3 test was applied. Survival curves were analyzed using the Kaplan–Meier method and compared by the log-rank test. All statistical analyses were performed using GraphPad Prism (version 8.0.1), with significance defined as *p <* 0.05 (*), *p <* 0.01 (**), and *p <* 0.001 (***). Non-significant differences (*p* ≥ 0.05) are denoted as “ns.”

## Results

### Highly fucosylated PvHMC preferentially associates with the ER chaperone PvBip

**Figure 1.**
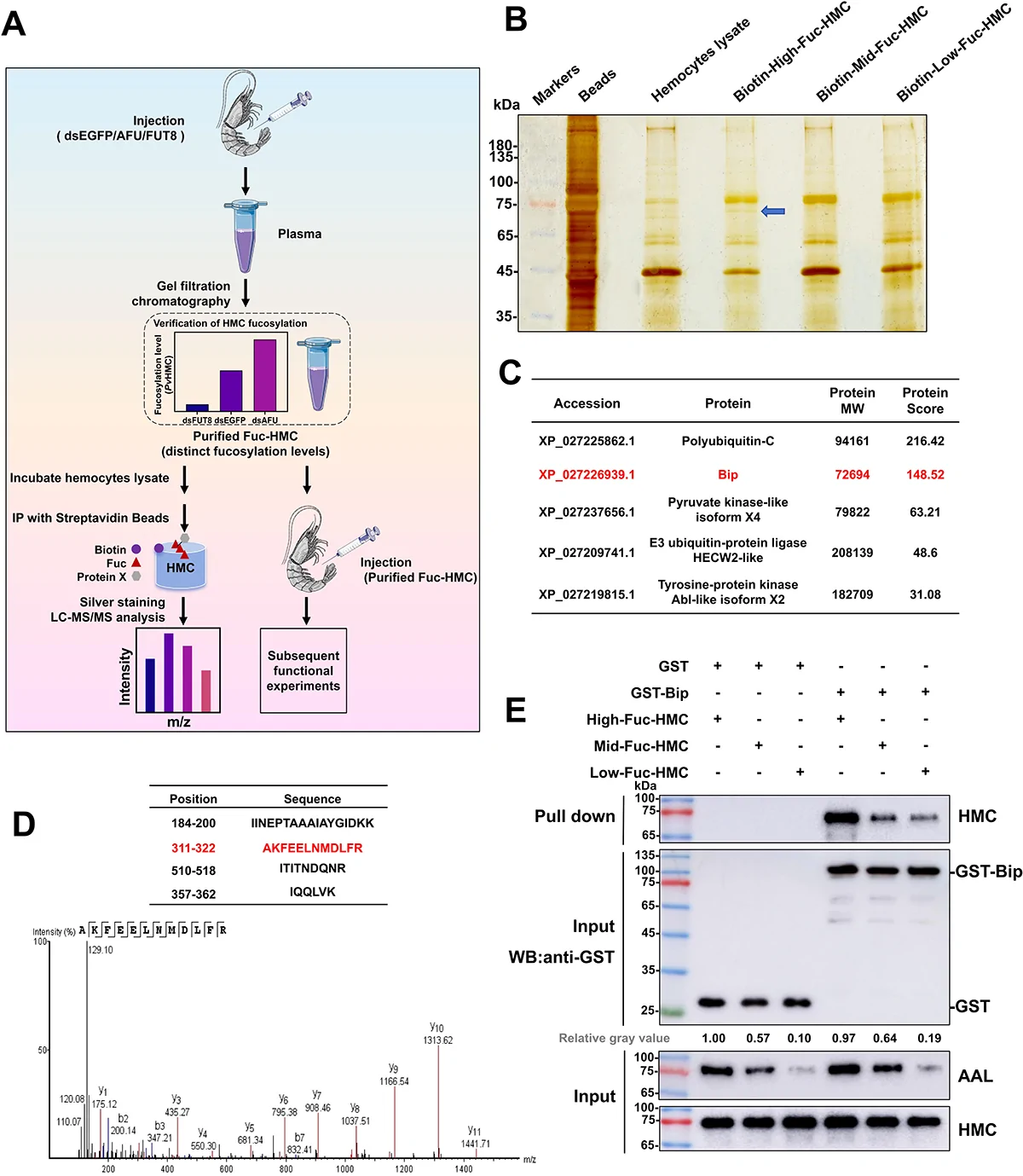
Identification and validation of proteins interacting with highly fucosylated *Pv*HMC. (A) Schematic workflow for the preparation of*Pv*HMC variants with different fucosylation levels, and subsequent interaction screening and functional analysis. (B) Silver staining of pulled-down proteins from hemocytes treated with*Pv*HMC of varying fucosylation levels. Arrowheads indicate distinct bands in pulled-down proteins from the High-Fuc-*Pv*HMC and Mid-Fuc-*Pv*HMC groups. (C) Top 5 proteins with the highest abundance values identified by MS. (D) Representative MS/MS spectra of*Pv*Bip-derived peptides. Four unique*Pv*Bip peptides were identified (top), with a representative MS/MS spectrum shown (bottom). (E)*In vitro*GST pull-down assay to verify interactions between*Pv*HMC (different fucosylation levels) and GST-tagged*Pv*Bip. Following treatment with fucosylated*Pv*HMC, immunoprecipitation (IP) was performed using anti-GST antibody, and immunoblotting was conducted with indicated antibodies. *Pv*HMC fucosylation levels in samples were assessed by Western blot and lectin blot with Aleuria aurantia lectin (AAL; 1:1000) and streptavidin-HRP (1:3000) (bottom). Data are representative of three independent experiments. All data are from three independent experiments with biological duplicates in each (*n* = 3) and shown as mean ± SEM.

To identify intracellular molecular partners of fucosylated *P. vannamei* hemocyanin (*Pv*HMC), we first generated and purified *Pv*HMC variants with distinct fucosylation levels through *in vivo* RNA interference followed by gel‑filtration chromatography (Figure 1(A)). Briefly, knockdown of *Pv*AFU yielded highly fucosylated *Pv*HMC (High-Fuc-HMC), injection of *Pv*EGFP dsRNA served as the moderate-fucosylation control (Mid-Fuc-HMC), and *Pv*FUT8 knockdown produced lowly fucosylated *Pv*HMC (Low-Fuc-HMC).

Biotin pull-down assays using High-Fuc-HMC as bait followed by silver staining of eluted protein complexes revealed a prominent ~72 kDa band in the High-Fuc-HMC group that was markedly weaker in the Mid-Fuc-HMC group (Figure 1(B)). Liquid chromatography-tandem mass spectrometry (LC-MS/MS) analysis of this band identified five candidate proteins with the highest abundance scores (Figure 1(C)). Among these candidates, binding immunoglobulin protein (*Pv*Bip) was prioritized for validation due to its established role as a core endoplasmic reticulum stress (ERS) chaperone and its predicted molecular weight matching the 72 kDa band (Figure 1(D)).

GST pull-down assays further supported a fucosylation-associated increase in *Pv*HMC-*Pv*Bip binding (Figure 1(E)). High-Fuc-HMC exhibited robust binding to recombinant GST-*Pv*Bip while Mid-Fuc-HMC and Low-Fuc-HMC showed minimal to no binding activity. No interaction was detected between any *Pv*HMC variant and empty GST protein and lectin blot analysis in the lower panel of Figure 1(E) verified the fucosylation status of each *Pv*HMC variant.

### Highly fucosylated PvHMC enhances a PvBip-PvXbp1s associated ER stress response in shrimp hemocytes

To determine if *Pv*HMC fucosylation modulates ERS activation, we quantified the expression of two canonical ERS markers (*Pv*Bip and the spliced transcription factor *Pv*Xbp1s) in hemocytes from shrimp treated with graded-fucosylation *Pv*HMC variants or an EGFP control.

**Figure 2.**
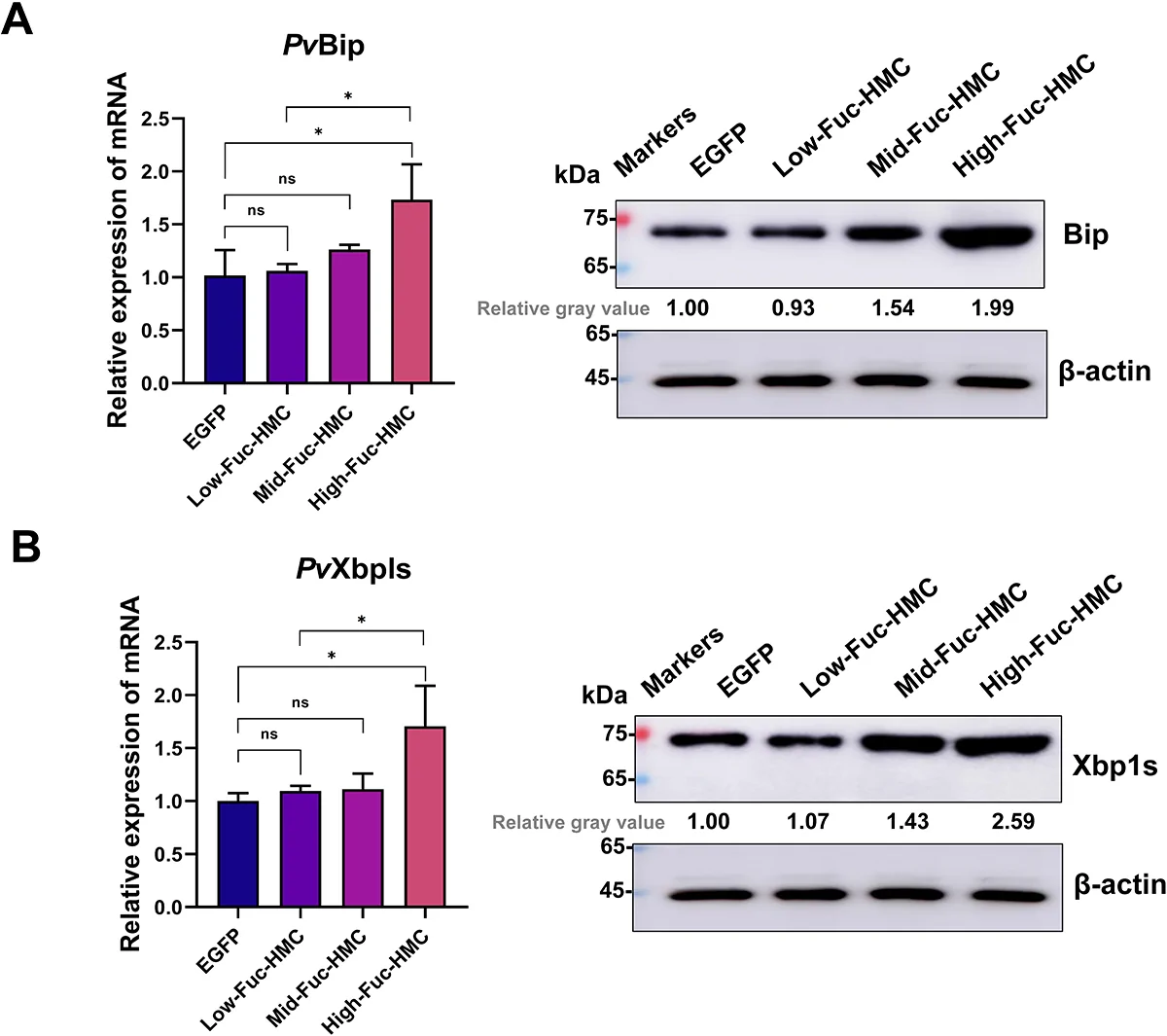
Fucosylated *Pv*HMC modulates endoplasmic reticulum (ER) stress-related protein expression in *P. vannamei* hemocytes. (A-B) mRNA and protein expression levels of (A)*Pv*Bip and (B)*Pv*Xbp1s in hemocytes treated with*Pv*HMC of varying fucosylation levels. mRNA levels were quantified by RT-qPCR and normalized to EF-1α. Protein levels were determined by Western blot using specific antibodies, with β-actin as the internal control. Band intensities were analyzed via ImageJ and normalized to β-actin. Results are presented as mean ± SEM (*n* = 3). *p < 0.05, **p < 0.01 vs control (EGFP). Statistical significance was assessed by one-way ANOVA followed by Tukey’s multiple-comparison test. Immunoblots are representative of at least three independent experiments.

Quantitative PCR and Western blot analyses demonstrated that High-Fuc-HMC treatment significantly upregulated *Pv*Bip at both mRNA and protein levels (Figure 2(A)). *Pv*Bip mRNA levels increased 1.73-fold relative to controls (*p* < 0.05), and corresponding protein levels rose 1.99-fold (*p* < 0.05). Mid-Fuc-HMC and Low-Fuc-HMC treatment had no significant impact on *Pv*Bip expression (all *p* > 0.05 vs control).

Consistent with *Pv*Bip expression trends, High-Fuc-HMC induced a 1.70-fold increase in *Pv*Xbp1s mRNA (*p* < 0.05) and a 2.59-fold increase in *Pv*Xbp1s protein (*p* < 0.05) relative to controls (Figure 2(B)). Neither Mid-Fuc-HMC nor Low-Fuc-HMC altered *Pv*Xbp1s expression (all *p* > 0.05 vs control). These data indicate that highly fucosylated *Pv*HMC, but not mid- or low-fucosylation *Pv*HMC, enhances *Pv*Bip and *Pv*Xbp1s expression in shrimp hemocytes.

### Fucosylated PvHMC promotes AMP expression through an ER stress associated PvXbp1s response

We first tested whether pharmacological modulation of ER stress affects AMP expression in *P. vannamei* hemocytes using the ERS activator tunicamycin (TM) and inhibitor 4-phenylbutyric acid (4-PBA). TM treatment significantly upregulated *Pv*Bip (Fig. S1A; *p <* 0.01) and *Pv*Xbp1s (Fig. S1B; *p <* 0.01) at both mRNA and protein levels while 4-PBA suppressed expression of both markers (Fig. S1A, C; *p <* 0.05). Concordantly, TM exposure increased transcription of *Pv*ALF2, *Pv*ALF3, *Pv*PEN2, and *Pv*LYZ2 by 2–3-fold (Fig. S1D–G; *p <* 0.05) and 4-PBA reduced expression of these AMPs by 30–50% relative to DMSO controls (Fig. S1D–G; *p <* 0.05).

**Figure 3.**
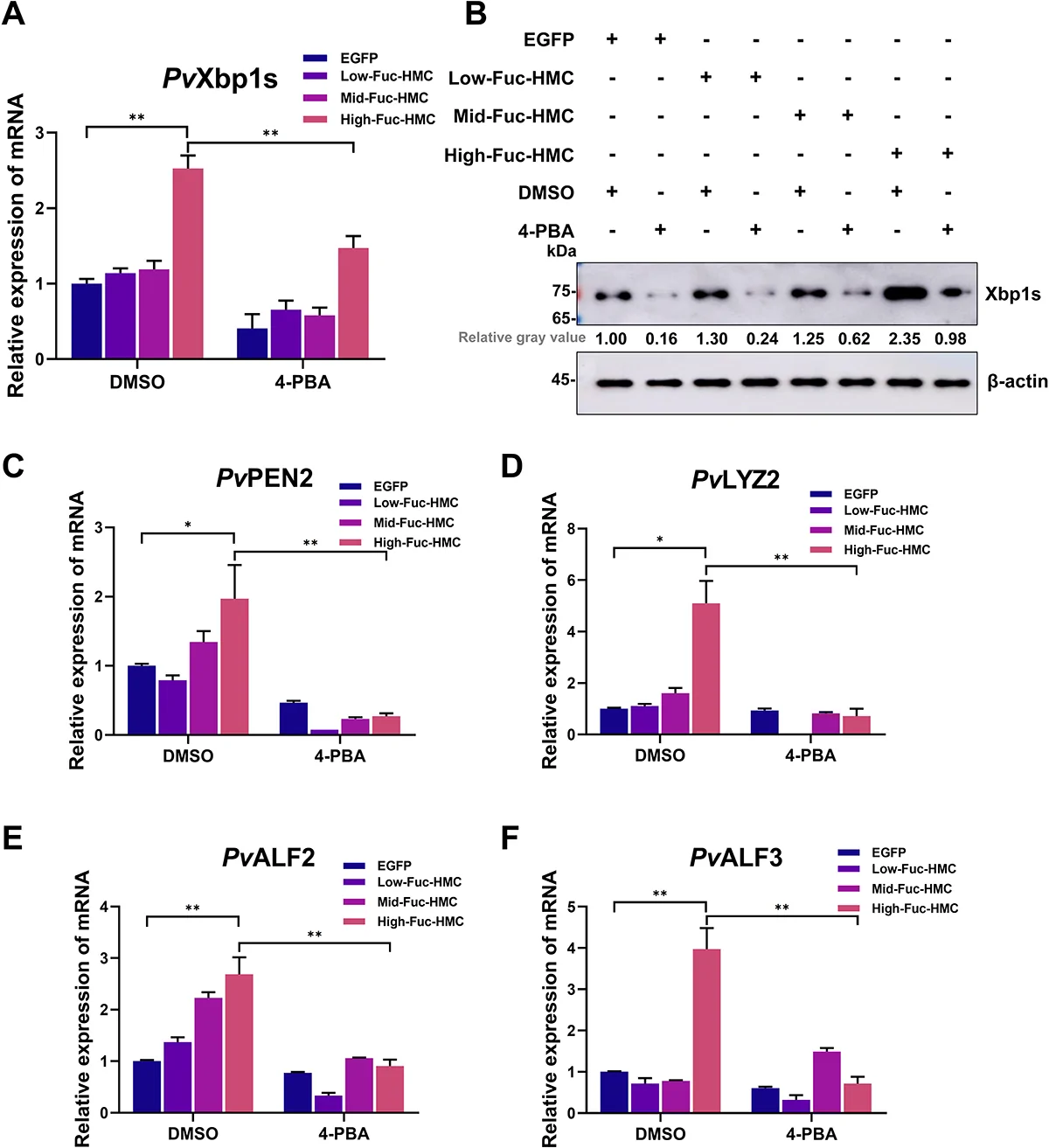
Fucosylated *Pv*HMC regulates antimicrobial peptide (AMP) expression via ER stress in *P. vannamei* hemocytes. (A-B) mRNA and protein expression levels of*Pv*Xbp1s in hemocytes treated with fucosylated*Pv*HMC (varying levels) with or without the ER stress inhibitor 4-PBA. (C-F) mRNA transcript levels of (C)*Pv*PEN2, (D)*Pv*ALF2, (E)*Pv*ALF3, and (F)*Pv*LYZ2 in hemocytes following the same treatment regimen. mRNA levels were quantified by RT-qPCR and normalized to EF-1α. Protein levels were determined by Western blot using specific antibodies, with β-actin as the internal control. Band intensities were analyzed via ImageJ and normalized to β-actin. Results are presented as mean ± SEM (*n* = 3). Statistical significance was assessed by one-way ANOVA followed by Tukey’s post hoc test (*p < 0.05; **p < 0.01). Immunoblots are representative of at least three independent experiments.

We then linked fucosylated *Pv*HMC to this axis focusing on *Pv*Xbp1s, a transcription factor with predicted binding motifs in the *Pv*PEN2 promoter (Table 2). To demonstrate direct transcriptional regulation, we performed RNAi-mediated knockdown of *Pv*Xbp1s. Compared with the dsEGFP control, ds*Pv*Xbp1s significantly reduced *Pv*Xbp1s expression (Fig. S2A; *p <* 0.01) and concomitantly decreased *Pv*PEN2 transcription (Fig. S2B; *p <* 0.05) in shrimp hemocytes. These data provide functional evidence that *Pv*Xbp1s is required for *Pv*PEN2 induction. Having confirmed that *Pv*Xbp1s acts as a key regulator of AMP expression, we next investigated whether its activation could be modulated by fucosylated *Pv*HMC. High-Fuc-HMC treatment elevated *Pv*Xbp1s mRNA levels (Figure 3(A); *p <* 0.01) and increased *Pv*Xbp1s protein abundance 2.35-fold relative to controls (Figure 3(B); *p <* 0.01). This treatment also induced significant upregulation of *Pv*PEN2 (3.2-fold), *Pv*LYZ2 (2.1-fold), *Pv*ALF2 (4.1-fold), and *Pv*ALF3 (3.8-fold) (Figure 3(C–F); *p <* 0.01). Mid-Fuc-HMC and Low-Fuc-HMC had no significant effect on *Pv*Xbp1s or AMP expression (all *p* > 0.05 vs control).

Co-treatment with 4-PBA markedly attenuated high-Fuc-HMC induced *Pv*Xbp1s upregulation (58% reduction in protein levels) (Figure 3(B); *p* < 0.05) and reversed AMP transcription to near-basal levels (Figure 3(C–F); *p* < 0.05 vs High-Fuc-HMC + DMSO). These results support the involvement of ER stress signaling in high-Fuc-HMC induced AMP expression.

### Fucosylated PvHMC enhances pathogen clearance and shrimp survival in an ER stress-dependent manner

**Figure 4.**
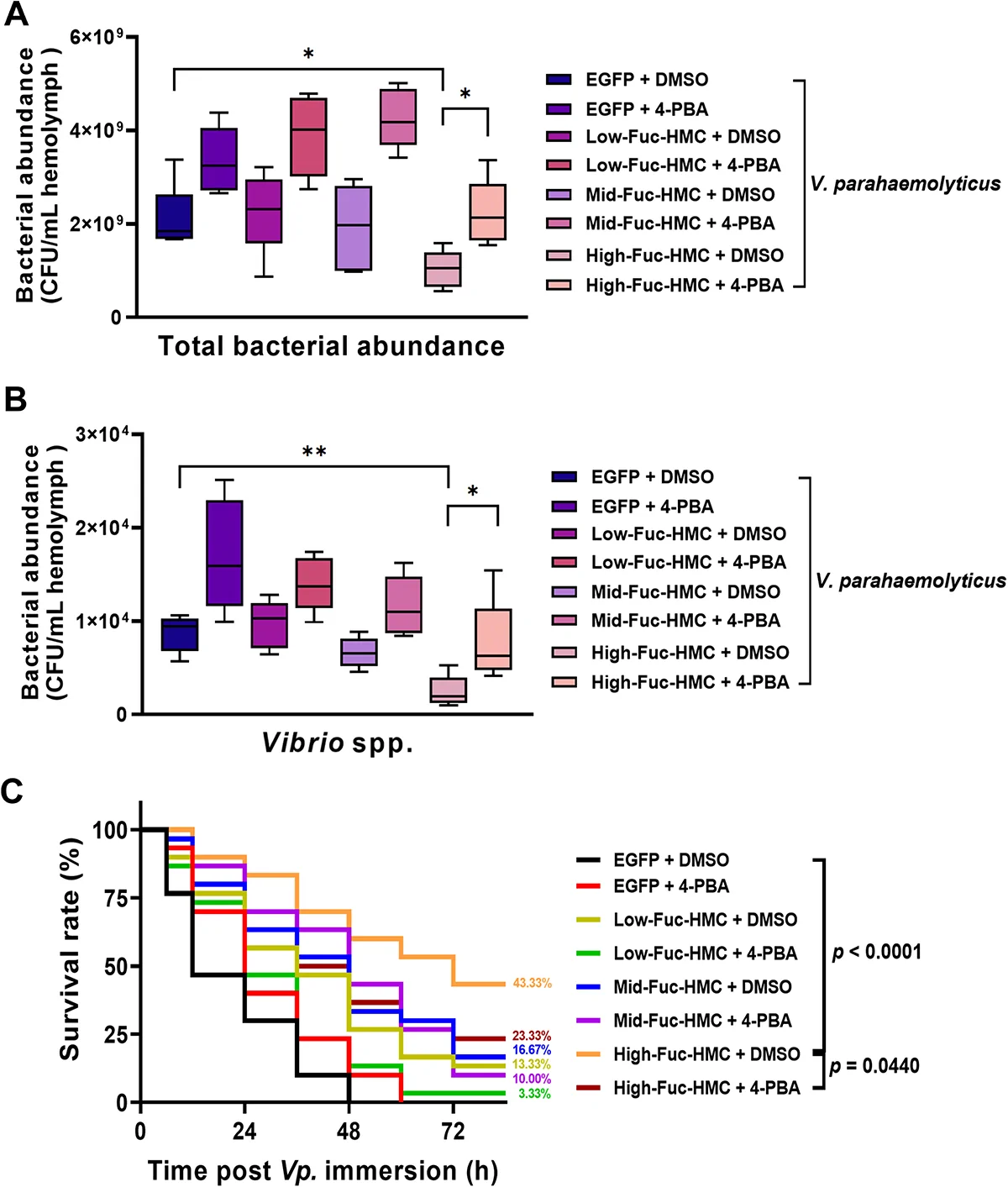
Fucosylated *Pv*HMC modulates hemolymph *V. parahaemolyticus* abundance and shrimp survival via ER stress. (A-B) Total bacterial abundance and relative *V. parahaemolyticus*abundance in shrimp hemolymph, determined by qPCR. Shrimp were injected with fucosylated*Pv*HMC (varying levels) with or without 4-PBA, followed by*V. parahaemolyticus*immersion challenge. Results are presented as mean ± SEM (*n* = 5). Statistical significance was assessed by two-way ANOVA followed by Sidak’s multiple-comparison test. (**p < 0.01; ns, not significant). (C) Kaplan–Meier survival curves of shrimp subjected to the same treatment and challenge regimen. Mortality was recorded every 12 h for 72 h. Survival curves were compared using the log-rank (Mantel-Cox) test, with*p* < 0.05 considered statistically significant.

To assess *in vivo* antibacterial function, we challenged shrimp with *V. parahaemolyticus* and quantified bacterial loads and survival across groups stratified by *Pv*HMC fucosylation level and ERS inhibition status. Total bacterial abundance in hemolymph was 60% lower in the High-Fuc-HMC + DMSO group than in the EGFP + DMSO control (Figure 4(A); *p <* 0.01). This protective effect was eliminated by 4-PBA co-treatment with bacterial loads returning to control levels (Figure 4(A); *p* > 0.05 vs control). Similarly, *Vibrio* levels were 70% reduced in High-Fuc-HMC + DMSO shrimp (Figure 4(B); *p <* 0.01) and 4-PBA restored *Vibrio* abundance to baseline (Figure 4(B); *p* > 0.05 vs control). Mid-Fuc-HMC and Low-Fuc-HMC treatment had no impact on bacterial or *Vibrio* clearance (all *p* > 0.05 vs control).

Survival assays over 72 h post-challenge showed that the High-Fuc-HMC + DMSO group had a cumulative mortality rate of 10.00% compared to 43.33% in EGFP + DMSO controls (Figure 4(C); log-rank test *p* = 0.0440). 4-PBA co-treatment increased mortality in High-Fuc-HMC shrimp to 23.33%, a rate comparable to that of Mid-Fuc-HMC (16.67%) and Low-Fuc-HMC (13.33%) groups (Figure 4(C); *p* > 0.05 between groups). In EGFP-treated infected shrimp, 4-PBA increased cumulative mortality to 33.33%, suggesting that ER stress inhibition compromised antibacterial resistance. Collectively, these data support a model linking *Pv*HMC fucosylation, ER stress‑associated AMP induction, and *in vivo* antibacterial defense.

## Discussion

As a multifunctional immune protein beyond oxygen transport [10–18], *Penaeus vannamei* hemocyanin (*Pv*HMC) is regulated by diverse post-translational modifications that collectively shape its antimicrobial activity. Our previous studies revealed that both fucosylation [24] and dephosphorylation [22] potentiate the immune functions of *Pv*HMC, indicating that this protein acts as a flexible immune hub rather than a static effector. Unlike our previous work, which mainly focused on the direct antibacterial activity of fucosylated *Pv*HMC, the present study identifies a *Pv*Bip-*Pv*Xbp1s associated ER stress response as a potential intracellular mechanism linking *Pv*HMC fucosylation to AMP induction and antibacterial defense.

Protein fucosylation is a conserved post-translational modification that modulates intermolecular interactions to govern diverse biological processes across taxa. Previous work has demonstrated that PoFUT1-mediated fucosylation enhances urokinase-type plasminogen activator binding to its receptor uPAR to activate RhoA-driven trophoblast angiogenesis [7] and regulates Notch receptor–ligand interactions to control downstream signaling cascades [34]. Fucosylation also modulates Wnt/β-catenin activity by altering low-density lipoprotein receptor-associated protein 6 endocytosis [35]. These studies have focused primarily on fucosylation-dependent regulation of developmental or oncogenic pathways, whereas its role in orchestrating invertebrate innate immune signaling remains largely uncharacterized. Our data help address this gap by identifying a novel interaction between fucosylated *Pv*HMC and the endoplasmic reticulum (ER) chaperone *Pv*Bip (Figure 1). This finding expands the known functional scope of protein fucosylation to include ERS-dependent antimicrobial immunity in crustaceans.

Bip acts as a master regulator of ER homeostasis by sequestering ERS transmembrane sensors under basal conditions and dissociating from these sensors to trigger ERS upon proteotoxic stress [36]. Previous reports have linked Bip interactions to ERS induction in pathological contexts: bacterial lipopolysaccharide drives Bip-p53 binding to exacerbate intestinal epithelial cell apoptosis [37] and amyloidogenic lysozyme variants associate with Bip to accumulate in the ER and promote cell death [38]. Our results extend this paradigm to innate immunity, showing that *Pv*HMC fucosylation enhances Bip binding to modulate ERS in shrimp hemocytes. This aligns with broader evidence that post-translational modifications tune ERS to execute immune functions, such as how reduced FOXO1 acetylation mitigates neuronal ERS-induced apoptosis to improve cognitive function in Alzheimer’s disease models [39]. These findings identify *Pv*HMC fucosylation as an important regulatory layer that connects hemocyanin modification with ER stress-associated immune signaling.

Aquatic invertebrates rely on a diverse repertoire of antimicrobial peptides (AMPs) including penaeidins [40,41], crustins [42,43], anti-lipopolysaccharide factors [44,45], and lysozymes [46,47] to mediate innate immunity, with AMP expression dynamics varying by pathogen stimulus and tissue type [48]. While ERS is a conserved regulator of immune responses in vertebrates, its role in driving AMP production in invertebrates had not been defined prior to our work. We demonstrate that ERS activation via tunicamycin upregulates key AMPs (*Pv*ALF2, *Pv*ALF3, *Pv*PEN2, *Pv*LYZ2) and ERS inhibition with 4-phenylbutyric acid (4-PBA) suppresses AMP transcription (Fig. S1). This finding suggests that ER stress can serve as a stress–immune interface in shrimp hemocytes.

X-box binding protein 1 (Xbp1) is a core ERS-responsive transcription factor whose spliced isoform (*Pv*Xbp1s) orchestrates transcriptional programs to restore ER homeostasis [49]. Our identification of high-confidence *Pv*Xbp1s binding motifs in the *Pv*PEN2 promoter (Table 2), combined with functional validation, supports a functional requirement for *Pv*Xbp1s in *Pv*PEN2 induction and suggests that *Pv*Xbp1s may contribute to AMP transcription through predicted promoter-binding motifs (Fig. S2). We further link this to *Pv*HMC fucosylation, showing that high-fucosylation *Pv*HMC (High-Fuc-HMC) elevates *Pv*Xbp1s levels to drive AMP transcription and that 4-PBA co-treatment abrogates this response (Figure 3). These data delineate a novel “fucosylated *Pv*HMC-ERS-*Pv*Xbp1s-AMP” axis, which provides, to our knowledge, evidence that a PTM-modified oxygen-transport protein can participate in ER stress-associated AMP regulation in an invertebrate.

Host-microbiota homeostasis is vital for physiological processes such as immune defense and host behavior [50,51]. In shrimp aquaculture systems, *Vibrio* species are ubiquitous opportunistic bacteria that may shift toward pathogenicity under environmental stress, causing severe disease outbreaks and mortality [52,53]. Our *in vivo* assays demonstrate that High-Fuc-HMC reduces hemolymph total bacterial and *V. parahaemolyticus* loads and improves shrimp survival post-challenge, while 4-PBA treatment reverses these protective effects (Figure 4(A,B)). Survival analysis further confirms that fucosylated *Pv*HMC enhances antimicrobial defenses via ERS modulation (Figure 4(C)), consistent with prior work showing reduced AMP expression correlates with increased shrimp mortality during pathogenic infection [54]. Notably, mannose modification appears to facilitate hemocyanin uptake and pathogen endocytosis [55], whereas the present study suggests that fucosylation contributes to *Pv*Bip‑associated ER stress signaling and AMP induction. Thus, hemocyanin glycosylation may not simply enhance direct antimicrobial activity, but may also diversify intracellular immune-regulatory functions.

**Figure 5.**
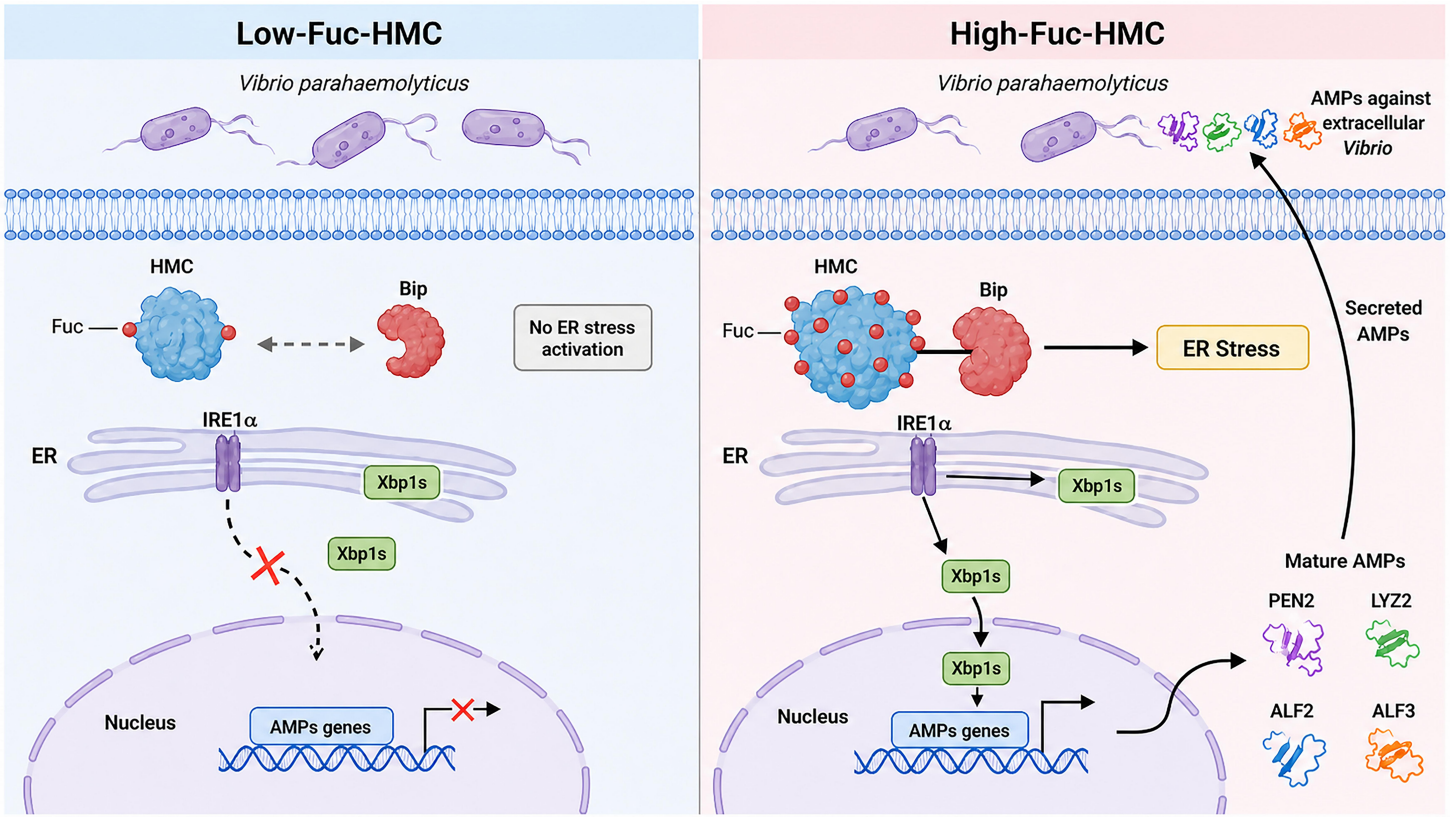
Proposed model of fucosylated hemocyanin-associated ER stress signaling in shrimp antibacterial immunity. In this proposed model,*Pv*HMC fucosylation enhances its association with*Pv*Bip and promotes*Pv*Bip-*Pv*Xbp1s-associated ER stress signaling. This response contributes to AMP induction and antibacterial defense during*V. parahaemolyticus*challenge.

This glycosylation-dependent regulation further supports the emerging view that hemocyanin functions as a highly adaptable immune hub rather than a static oxygen-transport protein. Across species, hemocyanin activity is shaped by multiple regulatory mechanisms, including fucosylation, mannosylation, dephosphorylation, deacetylation, and even electrostatic interactions that can promote the conversion of hemocyanin into phenoloxidase in horseshoe crabs [56]. Our findings identify fucosylation as a specific regulatory layer that links *Pv*HMC to *Pv*Bip-*Pv*Xbp1s‑associated ER stress signaling, AMP induction, and antibacterial defense during *V. parahaemolyticus* challenge (Figure 5). These results expand the known immune functions of hemocyanin from direct antimicrobial activity to ER stress-associated immune modulation. Moreover, this work provides a framework for understanding how PTMs diversify hemocyanin-mediated immune responses in crustaceans and suggests that fucosylation may represent a potential target for improving disease resistance in aquaculture species.

## Conclusion

In conclusion, this study demonstrates that hemocyanin fucosylation functions not only as a determinant of direct antibacterial activity but also as a regulator of intracellular stress-immune signaling in *P. vannamei*. Highly fucosylated *Pv*HMC preferentially associates with the ER chaperone *Pv*Bip, enhances *Pv*Bip-*Pv*Xbp1s-associated ER stress signaling, and promotes the transcription of key antimicrobial peptides, including *Pv*PEN2, *Pv*LYZ2, *Pv*ALF2, and *Pv*ALF3. Pharmacological inhibition of ER stress by 4-PBA attenuates AMP induction and compromises the protective effects of High-Fuc-PvHMC against *V. parahaemolyticus* challenge. These findings establish a previously unrecognized *Pv*HMC fucosylation-*Pv*Bip/*Pv*Xbp1s-AMP regulatory axis and expand the functional repertoire of hemocyanin from a classical oxygen-transport and immune effector protein to an intracellular modulator of PTM-dependent antibacterial signaling. This work provides new mechanistic insight into crustacean antibacterial immunity and suggests that hemocyanin fucosylation may represent a potential molecular target for improving disease resistance in shrimp aquaculture.

## Supplementary Material

Supplementary materials.docx

## Data Availability

The data that support the findings of this study are openly available in Mendeley Data at https://doi.org/10.17632/wbgnfbw5jt.1, reference number [57].
