# Supplementary material for: Fucosylated hemocyanin regulates ER stress to enhance shrimp antibacterial immunity
Source: Virulence. 2026 Sep 10;17(1):2721775. doi: 10.1080/21505594.2026.2721775 (PMC13568572; doi:10.1080/21505594.2026.2721775)
Supplement: Supplementary file 1 [file KVIR_A_2721775_SM5146.docx]

**Fig. S1. Endoplasmic reticulum (ER) stress modulators regulate antimicrobial peptide (AMP) expression in *P. vannamei* hemocytes.**

(A-C) The (A) mRNA and protein expression levels of *Pv*Bip, and *Pv*Xbp1s after treatment with ER stress (B) activator tunicamycin (TM) or (C) inhibitor 4-Phenylbutyric acid (4-PBA). (D-G) The mRNA transcript levels of (D) *Pv*CRUs, (E) *Pv*ALFs, (F) *Pv*PENs, and (G) *Pv*LYZs in shrimp hemocytes after treatment with tunicamycin (TM) or 4-Phenylbutyric acid (4-PBA). Control samples were treated with 6% diluted dimethyl sulfoxide (DMSO). The mRNA levels were quantified by RT-qPCR and normalized to those of EF-1a mRNA, while protein levels were determined by Western blot using the indicated antibodies, with β-actin used as the loading control. Protein band intensity was analyzed using ImageJ and normalized relative to β-actin. Results are reported as mean ± SEM (n = 3). * *p <* 0.05, ** *p <* 0.01 vs. control (DMSO). The immunoblots shown are representative of at least three independent experiments.

**Fig. S2. RNAi-mediated knockdown of *Pv*Xbp1s reduces *Pv*PEN2 expression in *P. vannamei* hemocytes.**

(A-B) mRNA transcript levels of (A) *Pv*Xbp1s and (B) *Pv*PEN2 in hemocytes after RNAi-mediated knockdown of *Pv*Xbp1s. mRNA levels were quantified by RT-qPCR and normalized to EF-1α. Results are presented as mean ± SEM (n = 3). **p* < 0.05, ***p* < 0.01 vs. control (EGFP) (unpaired Student’s *t*-test).
